# Tumor Prognostic Prediction of Nasopharyngeal Carcinoma Using CT-Based Radiomics in Non-Chinese Patients

**DOI:** 10.3389/fonc.2022.775248

**Published:** 2022-01-28

**Authors:** Sararas Intarak, Yuda Chongpison, Mananchaya Vimolnoch, Sornjarod Oonsiri, Sarin Kitpanit, Anussara Prayongrat, Danita Kannarunimit, Chakkapong Chakkabat, Sira Sriswasdi, Chawalit Lertbutsayanukul, Yothin Rakvongthai

**Affiliations:** ^1^Medical Physics Program, Department of Radiology, Faculty of Medicine, Chulalongkorn University, Bangkok, Thailand; ^2^Chulalongkorn University Biomedical Imaging Group, Department of Radiology, Faculty of Medicine, Chulalongkorn University, Bangkok, Thailand; ^3^Biostatistics Excellence Center, Research Affairs, Faculty of Medicine, Chulalongkorn University, Bangkok, Thailand; ^4^Division of Radiation Oncology, Department of Radiology, King Chulalongkorn Memorial Hospital, Bangkok, Thailand; ^5^Division of Radiation Oncology, Department of Radiology, Faculty of Medicine, Chulalongkorn University, Bangkok, Thailand; ^6^Research Affairs, Faculty of Medicine, Chulalongkorn University, Bangkok, Thailand; ^7^Center for Artificial Intelligence in Medicine, Faculty of Medicine, Chulalongkorn University, Bangkok, Thailand; ^8^Division of Nuclear Medicine, Department of Radiology, Faculty of Medicine, Chulalongkorn University, Bangkok, Thailand

**Keywords:** nasopharyngeal carcinoma, prognosis, radiomics, imaging biomarkers, computed tomography

## Abstract

**Purpose:**

We aimed to construct predictive models for the overall survival (OS), progression-free survival (PFS), and distant metastasis-free survival (DMFS) for nasopharyngeal carcinoma (NPC) patients by using CT-based radiomics.

**Materials and Methods:**

We collected data from 197 NPC patients. For each patient, radiomic features were extracted from the CT image acquired at pretreatment *via* PyRadiomics. Feature selection was performed in two steps. First, features with high inter-observer variability based on multiple tumor delineations were excluded. Then, stratified bootstrappings were performed to identify feature combinations that most frequently achieved the highest (i) area under the receiver operating characteristic curve (AUC) for predicting 3-year OS, PFS, and DMFS or (ii) Harrell’s C-index for predicting time to event. Finally, regularized logistic regression and Cox proportional hazard models with the most frequently selected feature combinations as input were tuned using cross-validation. Additionally, we examined the robustness of the constructed model to variation in tumor delineation by simulating 100 realizations of radiomic feature values to mimic features extracted from different tumor boundaries.

**Results:**

The combined model that used both radiomics and clinical features yielded significantly higher AUC and Harrell’s C-index than models using either feature set alone for all outcomes (*p* < 0.05). The AUCs and Harrell’s C-indices of the clinical-only and radiomics-only models ranged from 0.758 ± 0.091 to 0.789 ± 0.082 and from 0.747 ± 0.062 to 0.767 ± 0.074, respectively. In comparison, the combined models achieved AUC of 0.801 ± 0.075 to 0.813 ± 0.078 and Harrell’s C-indices of 0.779 ± 0.066 to 0.796 ± 0.069. The results showed that our models were robust to variation in tumor delineation with the coefficient of variation ranging from 4.8% to 6.4% and from 6.7% to 9.3% for AUC and Harrell’s C-index, respectively.

**Conclusion:**

Our results demonstrated that using CT-based radiomic features together with clinical features provided superior NPC prognostic prediction than using either clinical or radiomic features alone.

## 1 Introduction

Nasopharyngeal carcinoma (NPC) is one of the most malignant head and neck cancers worldwide and is endemic in Southeast Asia and Southern China (1, 2). According to the International Agency for Research on Cancer, there were about 130,000 new cases of nasopharyngeal carcinoma in 2020 (3). The mainstay treatment of NPC is intensity-modulated radiation therapy (IMRT) with chemotherapy. However, the treatment strategies, e.g., concurrent chemoradiotherapy (CCRT), CCRT followed by adjuvant chemotherapy, or induction chemotherapy followed by CCRT need personalized data for therapeutic planning (4).

In NPC, clinical staging according to the American Joint Committee on Cancer (AJCC) is conventionally used to guide the optimal treatment and determine cancer prognosis (5–8). Until recently, plasma Epstein–Barr virus (EBV) DNA has been widely used for early detection, prognostication, and monitoring of treatment response of NPC (8–12). Nevertheless, the EBV DNA level was undetectable up to 40% in non-Chinese series (13). Therefore, an additional effective biomarker is needed to improve the prognostic performance.

Medical imaging is a routine practice in oncologic patient management for tumor diagnosis and staging, treatment planning, and response monitoring. Computed tomography (CT) and magnetic resonance imaging (MRI) are common modalities in NPC imaging. Tumor appearance in radiographic images and demographic features such as patients’ age, tumor stage, and performance status were used to select an appropriate treatment. However, conventionally, the radiologic tumor description is provided qualitatively by focusing on tumor size and anatomical extension without considering intratumoral heterogeneity, which is predictive of the prognostic outcomes (14, 15). Radiomics, an emerging technique for tumor characterization, is the process of converting medical images into minable high-dimensional data (16–19). It refers to the extraction of quantitative features, so-called radiomic features, which describe detailed tumor characteristics, from whole tumor mass. Examples of radiomic features include tumor size, geometry, voxel intensity, and texture patterns. Several studies showed potential of radiomic features in head and neck cancer prognostic prediction. Aerts et al. (20) found that the radiomics model was able to capture intratumoral heterogeneity and was significantly associated with the gene-expression profile pattern. Moreover, results from various studies showed high correlation between radiomic features and prognostic outcomes, such as in head and neck cancer (21, 22), esophageal cancer (23, 24), and nasopharyngeal cancer (25, 26).

In NPC, several studies on radiomics also showed great promise for prognosis. Du et al. (25) used pretreatment contrast-enhanced T1- or T2-weighted MRI to construct predictive models for 3-year disease progression after IMRT treatment. The results showed that combining radiomics with other clinical features including age, sex, and TNM staging yielded higher prognostic performance than using clinical features alone. Similarly, Ming et al. (26) found that combining clinical features with MRI-based radiomics yielded higher performance. Some studies integrated pretreatment Epstein–Bar virus (EBV) DNA information as part of clinical features (27–29). The combined model of clinical and radiomic features yielded the highest prognostic performance in these studies as well.

In countries with limited resources, CT scans are more widely used and accessible than MRI because they are typically less expensive. However, while there were several research studies that used MRI data of non-metastasis NPC patients, none of them investigated the predictive value of CT-based radiomics in NPC. Therefore, in our study, we used CT-based radiomics to build the predictive model for NPC. We hypothesized that CT information, like MRI’s, should also improve the predictive performance. Moreover, if successful, our model would be beneficial for centers without an MRI simulator.

Specifically, we developed prognostic models for NPC based on conventional clinical features, pretreatment CT radiomic features, and the combination of the two. We compared their prognostic performances in terms of overall survival (OS), progression-free survival (PFS), and distant metastasis-free survival (DMFS) at 3 years as well as time-to-event outcomes.

## 2 Materials and Methods

### 2.1 Dataset

Data from newly diagnosed NPC patients at King Chulalongkorn Memorial Hospital, Thai Red Cross Society, Bangkok, Thailand, between October 2010 and September 2015 were collected. This group of patients was part of the patients who were enrolled in a previously reported randomized study comparing the IMRT technique between sequential versus simultaneous integrated boost technique (30). This study was approved by the Institutional Review Board of Faculty of Medicine, Chulalongkorn University, Bangkok, Thailand (IRB no. 745/61). The inclusion criteria of this study were as follows: (a) newly diagnosed with NPC patients, (b) no evidence of distant metastasis, (c) at least 3-year follow-up time, (d) underwent CT and MRI simulation, (e) received the IMRT with chemotherapy, and (f) available pretreatment plasma Epstein–Barr virus (EBV) DNA level. The clinical data included sex, age, and tumor staging. Patient were restaged according to the 8^th^ edition of the American Joint Committee on Cancer (AJCC) TNM staging system (5). Patients with more than T1 or positive nodal disease received IMRT 70 Gy in 33–35 fractions with a concurrent chemotherapy by weekly cisplatin 40 mg/m^2^ for a maximum of seven cycles. Cisplatin 80 mg/m^2^ and 5-fluorouracil (5-FU) 1,000 mg/m^2^/24 h were given as adjuvant chemotherapy over a 96-h continuous infusion at 4-week intervals for three cycles.

### 2.2 Region of Interest Segmentation

For each patient, the gross tumor volume (GTV) of the primary tumor in the nasopharynx was contoured on CT images by the treating radiation oncologist. When a GTV was drawn, the co-registered MR image was also presented. Of 197 patients, thirty patients were randomly selected and their GTVs were additionally contoured by two radiation oncologists. This created a multiple tumor delineation dataset (with 3 GTVs for each of these 30 patients) for testing inter-observer variability.

### 2.3 Model Construction

#### 2.3.1 Radiomic Feature Extraction

To ensure the spatial consistency in the radiomics analysis, all CT images were resampled into 0.5 × 0.5 × 3 mm^3^ voxels. A total of 842 radiomic features per GTV were calculated *via* Pyradiomics version 2.0.0 (31). The extracted features were classified into four feature classes (20), including the shape-based class, first-order intensity class, texture-based class, and wavelet-based class.

#### 2.3.2 Radiomic Feature Selection

After feature extraction, the number of features was reduced *via* a feature selection process to prevent model overfitting. To select robust and informative features, we performed inter-observer variability test and univariate analysis as follows.

##### 2.3.2.1 Inter-Observer Stability Test

The multiple delineation dataset was used for the inter-observer variability test. Good radiomic features should be consistent regardless of the radiation oncologist who drew the contour. The intra-class correlation (ICC) analysis was used to assess the correlation of features from multiple regions of interest (ROIs). According to a study of Koo et al. (32), features whose ICC values were less than 0.5 were excluded from further analyses.

##### 2.3.2.2 Feature Performance Analysis

To assess the feature’s performance, a univariable analysis using logistic regression and Cox proportional hazard regression analysis for the binary outcome [having survived (OS), being disease-free (PFS), and being DMFS at 3 years] and survival outcomes, respectively, was performed. We repeatedly sampled 80% of the dataset with stratification for 100 times to calculate the area under the receiver operating characteristic (ROC) curve (AUC) and Harrell’s C-index. For each repeat, each outcome, and each radiomic feature class (20), the top single feature that yielded the highest AUC or C-index on the training set was selected. The most frequently selected set of 4 radiomic features was chosen for subsequent radiomics model construction.

#### 2.3.3 Clinical Feature Selection

All clinical features including age, sex, T-stage, N-stage, overall stage (AJCC 8^th^ edition), and pretreatment plasma EBV DNA level (cutoff = 2,300 copies/ml) (13) were initially included into the multivariate logistic regression or Cox proportional hazard models. Then, backward feature elimination was performed to repeatedly remove unimportant features with Wald test *p*-values greater than 0.1 until no feature can be removed. This process was repeated 100 times by sampling 80% of the dataset with stratification. The most frequently retained set of clinical features were selected for subsequent clinical-only model constructions.

For the combined models which used both clinical and radiomic features, backward feature elimination of clinical features was performed similarly as described, with two additional constraints: the set of 4 radiomic features selected above were always retained in the models, and at least one clinical feature must remain in each model. The most frequently retained set of clinical features were selected for subsequent combined model constructions.

#### 2.3.4 Prognostic Model Construction

**Figure 1:**
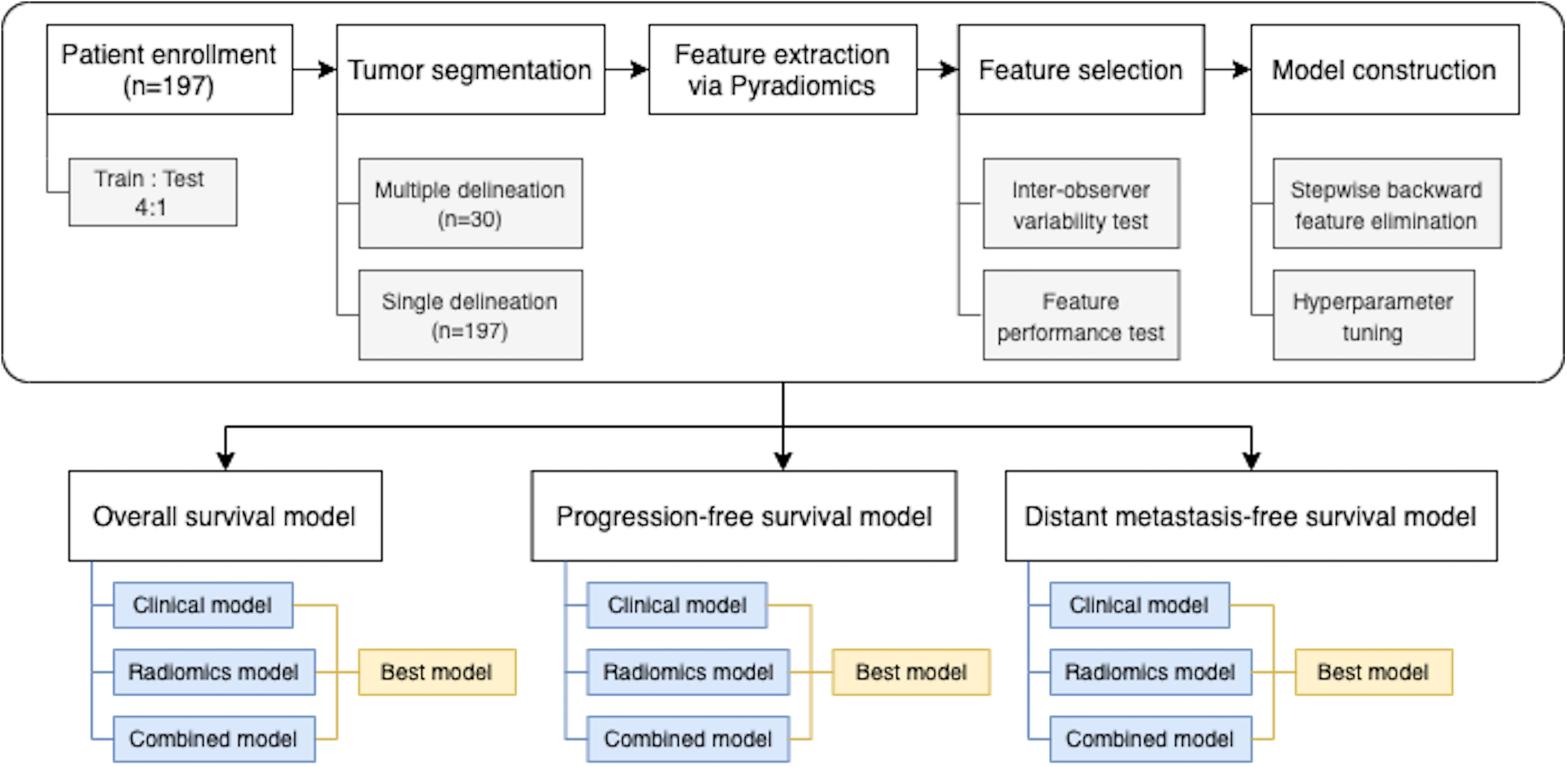
Overall process in this study.

For binary outcomes, which were 3-year OS, 3-year PFS, and 3-year DMFS, logistic regression models were built. L1 (Lasso) or L2 (Ridge) regularizations with inverse strength ranging from 0.001 to 10 were considered. For time-to-event outcomes, Cox proportional hazard regression models were used. Elastic net regularizations with strength ranging from 0.001 to 10 and L1/L2 ratio ranging from 0.001 to 1 were considered. The best hyperparameters were selected based on AUC or Harrell’s C-index from 20 realizations of 5-fold cross-validation (for a total of 100 repeats). Regularized model developments were performed in Python using the LogisticRegression module of scikit-learn library and the CoxPHFitter module of lifelines library (33, 34). The overall process of model construction is shown in the **Figure 1**.

### 2.4 Model Comparison (Statistical Analysis)

The sign test in the scipy package with default settings was used to compare the Radiomics, Clinical, and Combined models and the model that used only tumor volume. The Benjamini/Hochberg procedure was used for multiple-comparison correction. We also constructed the Kaplan–Meier plot for 100 repeats of the test set, where patients were classified as high risk if their predicted scores were higher than the median and vice versa. All statistical analyses, except sign test and log-rank test which were analyzed in Python, were performed using STATA version 15.0 (StataCorp LLC, Texas, USA) (35).

### 2.5 Testing Model Robustness to Variation in Radiomic Feature Extraction

**Figure 2:**
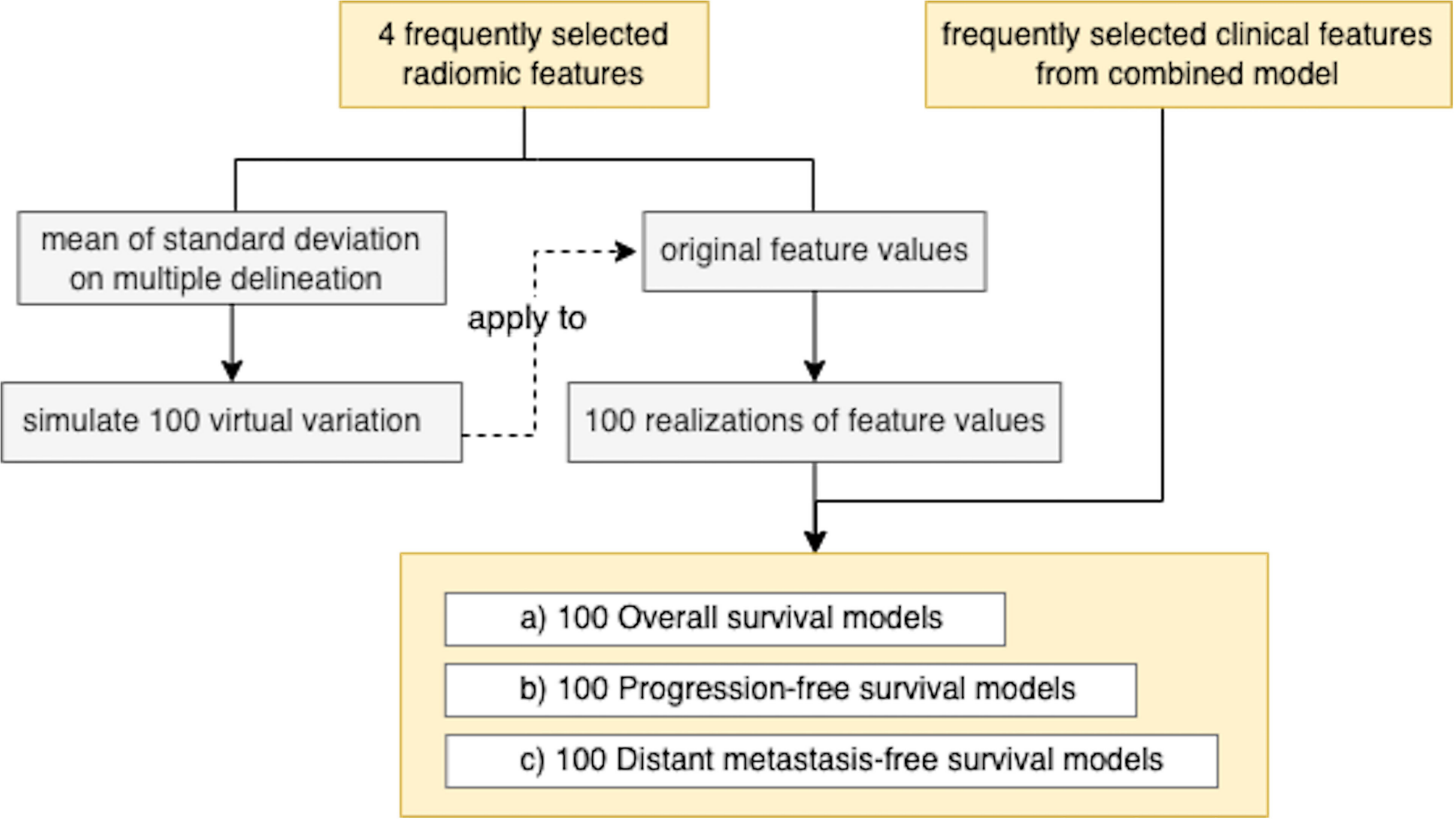
Process of model robustness to radiomic feature variation due to segmentation.

To investigate the model’s robustness to the variation in tumor delineation by different radiation oncologists, we generated 100 realizations of a normal random vector whose mean was equal to the radiomic features extracted and whose variance was equal to the sample variance calculated on the multiple-delineation dataset. For each outcome (OS/PFS/DMFS and binary/time-to-event), we computed the mean and standard deviation of the corresponding AUC or Harrell’s C-index across 100 realizations (**Figure 2**).

## 3 Results

### 3.1 Patient Characteristics

A total of 197 patients had non-metastatic nasopharyngeal cancer, and approximately 80% of them were classified as undifferentiated carcinoma (**Table 1**). For tumor (T) classification, the proportion of patients was 22.3%, 37.1%, 24.4%, and 16.2% for T1, T2, T3, and T4, respectively. For node (N) classification, the proportion was 3.0%, 24.4%, 49.2%, and 23.4% for N0, N1, N2, and N3, respectively, while for stage grouping, the proportion was 0.5%, 14.2%, 49.2%, and 36.1% for stages I, II, III, and IVA, respectively. Fifty percent of patients had pretreatment plasma EBV DNA level ≥2,300 copies/ml. The median age was 50 years (interquartile range: 43 to 56), and 79.2% were male. The median follow-up time was 4.9 years.

**Table 1:** Patient characteristics.

Characteristics	n = 197 patients (%)
Median age (years) (IQR)	50 (43 to 56)
Sex	
Male	156 (79.2)
Female	41 (20.8)
T classification	
T1	44 (22.3)
T2	73 (37.1)
T3	48 (24.4)
T4	32 (16.2)
N classification	
N0	6 (3.0)
N1	48 (24.4)
N2	97 (49.2)
N3	46 (23.4)
Stage group	
I	1 (0.5)
II	28 (14.2)
III	97 (49.2)
IVA	71 (36.1)
Pretreatment plasma EBV DNA level	
Undetectable or < 2300 copies/ml	100 (50.76)
≥2,300 copies/ml	97 (49.24)
Median EBV value (copies/ml) (IQR)	7,795 (3150 to 18000)
Pathologic classification	
Undifferentiated carcinoma	159 (80.71)
Differentiated non keratinizing carcinoma	37 (18.78)
Poorly differentiated squamous cell carcinoma	1 (0.51)

### 3.2 Radiomics Model

Out of 842 features, we found that 384 features passed the ICC criterion for the inter-observer stability test. Across 100 bootstrap repeats, the set of four features (one for each class) which was most frequently selected for each type of outcome is listed in **Table 2**. Interestingly, “original_firstorder_Uniformity” from the first-order class was consistently selected in all binary and time-to-event outcomes. For the texture class, “original_glfm_DependenceNonUniformity” was selected for OS outcomes while “original_glrlm_GrayLevelNonUniformity” was selected for PFS and DMFS outcomes.

**Table 2:** Most frequently selected features of OS, PFS, and DMFS in each class on NPC patients.

Statistical analysis	Feature group	Feature name		
		OS	PFS	DMFS
**Logistic regression** **(binary outcome)**	Shape	original_shape_MajorAxisLength	original_shape_MajorAxisLength	original_shape_MajorAxisLength
	First order	original_firstorder_Uniformity	original_firstorder_Uniformity	original_firstorder_Uniformity
	Texture	original_gldm_DependenceNonUniformity	original_glrlm_GrayLevelNonUniformity	original_glrlm_GrayLevelNonUniformity
	Wavelet	wavelet-LHL_gldm_LargeDependenceEmphasis	wavelet-LHL_glrlm_GrayLevelNonUniformity	wavelet-HHL_ngtdm_Busyness
	Clinical	age	age	age
		plasma EBV DNA level	plasma EBV DNA level	plasma EBV DNA level
**Cox regression** **(time-to-event outcome)**	Shape	original_shape_MajorAxisLength	original_shape_SurfaceArea	original_shape_SurfaceArea
	First order	original_firstorder_Uniformity	original_firstorder_Uniformity	original_firstorder_Uniformity
	Texture	original_gldm_DependenceNonUniformity	original_glrlm_GrayLevelNonUniformity	original_glrlm_GrayLevelNonUniformity
	Wavelet	wavelet-LHL_glrlm_RunVariance	wavelet-HLL_glrlm_RunLengthNonUniformity	wavelet-HLL_glrlm_RunLengthNonUniformity
	Clinical	age	age	age
		plasma EBV DNA level	plasma EBV DNA level	plasma EBV DNA level
				N stage (8th edition)

### 3.3 Clinical Model

The results from 100 bootstraps showed that the models for both binary and time-to-event outcomes selected the same feature sets. For OS and PFS outcomes, frequently selected clinical features were age, T-stage, and pretreatment EBV DNA level. For DMFS, age, T stage, N stage, and pretreatment EBV DNA level were selected.

### 3.4 Combined Model

We found that age and pretreatment EBV DNA level were commonly selected across all outcomes in the combined models. Furthermore, N stage was also selected in the model for the time-to-event DMFS outcome (**Table 2**).

### 3.5 Classification and Evaluation

**Figure 3:**
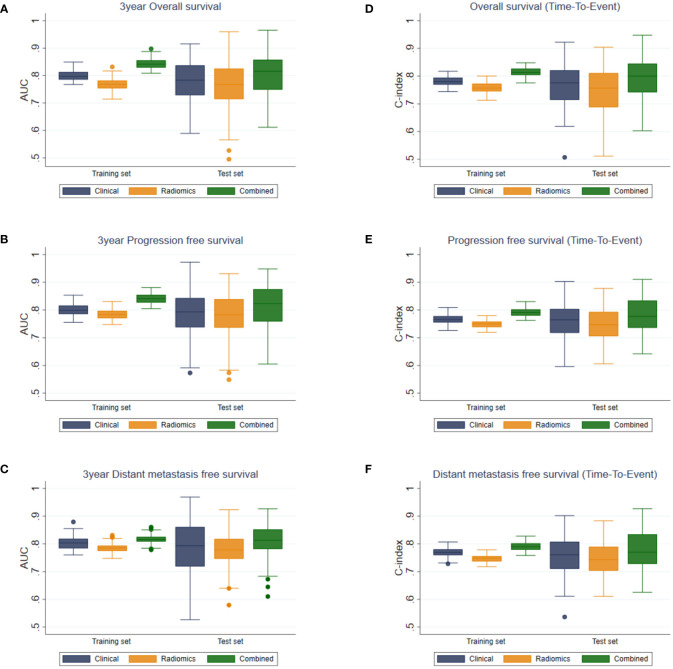
Boxplots of AUC values for **(A)** OS, **(B)** PFS, **(C)** DMFS, and C-indices for **(D)** OS, **(E)** PFS, and **(F)** DMFS of the clinical, radiomics, and combined models.

The results of multivariate analyses showed that the combined model yielded the best performance in every outcome as compared to the clinical and the radiomics models (**Figure 3**).

For binary outcome predictions, the combined model yielded AUCs of 0.817 ± 0.016 to 0.844 ± 0.018 and 0.801 ± 0.075 to 0.813 ± 0.078 on the training and test sets, respectively. In comparison, the AUCs of the clinical and radiomics models ranged from 0.769 ± 0.023 to 0.804 ± 0.024 on the training set and from 0.758 ± 0.091 to 0.789 ± 0.082 on the test set.

Harrell’s C index from the combined model in predicting time-to-event outcomes ranged from 0.791 ± 0.015 to 0.815 ± 0.017 and from 0.779 ± 0.066 to 0.796 ± 0.069 on the training and the test sets, respectively. These were higher than those from the clinical (0.767 ± 0.016–0.781 ± 0.017 and 0.756 ± 0.066–0.767 ± 0.074) and the radiomics models (0.747 ± 0.014–0.759 ± 0.018 and 0.747 ± 0.062–0.749 ± 0.077) on both the training and test sets. The performances of the combined model were significantly higher than those of the clinical or the radiomics model for all outcomes (*p* < 0.05).

The tumor volume yielded AUCs of 0.746 ± 0.024 to 0.773 ± 0.017 and 0.747 ± 0.097 to 0.776 ± 0.083 on the training and test sets, respectively. Harrell’s C index from the tumor volume ranged from 0.731 ± 0.022 to 0.744 ± 0.014 and from 0.734 ± 0.099 to 0.746 ± 0.064 on the training and test sets, respectively. The tumor volume yielded significantly lower AUC and Harrell’s C-index than those of the combined model for all outcomes (*p* < 0.001). Comparison of the tumor volume with the radiomics model in the test sets showed no significant difference between them in terms of AUC in OS, PFS, and DMFS predictions, and in terms of C-index in DMFS. For the other outcomes (OS and PFS predictions based on Harrell’s C-index), the radiomics model yielded higher performance metrics than the tumor volume with *p* < 0.05.

**Figure 4:**
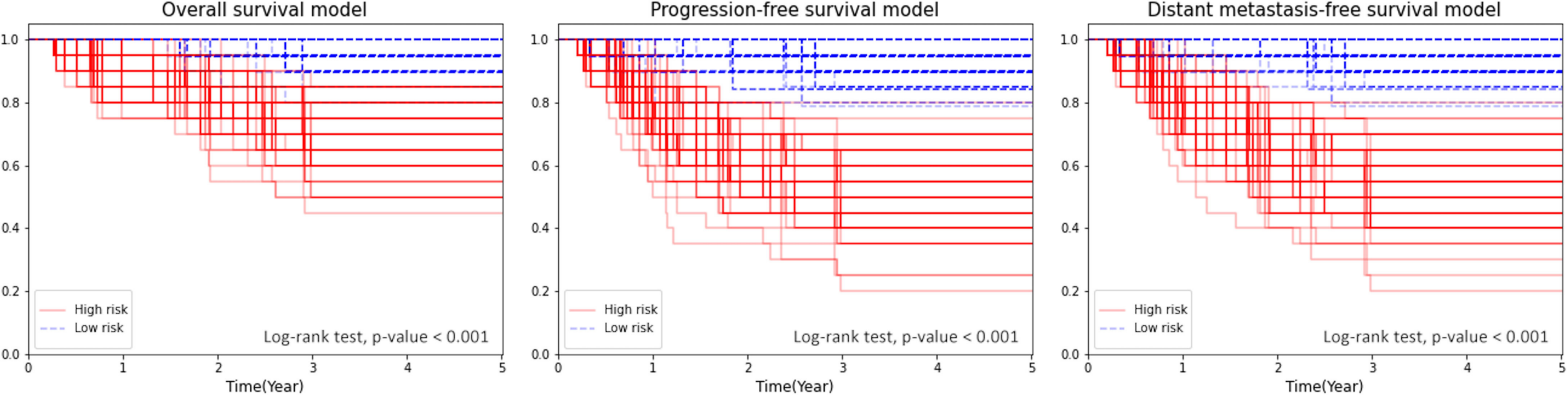
Kaplan–Meier plot for OS, PFS, and DMFS stratified by the median of the combined model’s score.

The Kaplan–Meier plot for the test sets showed a significant separation (log-rank test *p* < 0.001 in all 100 repeats) between the survival curves of the high-risk and low-risk groups, which were defined by the median of predictions as described in Section 2.4 (**Figure 4**).

### 3.6 Model Robustness to Variation in Radiomic Feature Extraction

After adding uncertainty to the radiomic feature values and reconstructing the models, we found that the mean and standard deviation among 100 computed AUC values were 0.886 ± 0.043, 0.818 ± 0.052, and 0.781 ± 0.047 in OS, PFS, and DMFS, respectively. On the other hand, Harrell’s C-indices were 0.860 ± 0.064, 0.757 ± 0.070, and 0.819 ± 0.055 in OS, PFS, and DMFS, respectively. The corresponding coefficient of variation ranged from 4.8% to 6.4% and from 6.7% to 9.3% for AUC and Harrell’s C-index, respectively.

## 4 Discussion

Clinical data such as TNM staging (5), age, and blood test (EBV concentration level) were traditionally used for predicting prognosis in NPC patients and determining treatment strategy. Based on Lertbutsayanukul et al. (13), the optimal cutoff value for plasma EBV DNA for predicting OS, PFS, and DMFS was 2,300 copies/ml. Hence, the same cutoff was applied in our analysis. This transformed the pretreatment plasma EBV DNA level into a discrete variable. In our study, we constructed NPC tumor prognostic models using various combinations of CT-based radiomic features and clinical features. The corresponding AUC and Harrell’s C-index of OS, PFS, and DMFS outcomes for each constructed model were used to compare performances and identify the best models.

The combined model incorporating both clinical and radiomic features was superior to the clinical or radiomics model for OS, PFS, and DMFS predictions. This demonstrated the predictive value of quantitative information extracted from medical images when used in conjunction with conventional clinical features. Beyond the volumetric measurement, radiomic features also described tumor characteristics, especially intratumor heterogeneity, which has been shown to be related to tumor prognosis.

In our predictive models, we found that the most frequently selected radiomic features from the shape class were “original_shape_MajorAxisLength” and “original_shape_SurfaceArea.” Features in the shape class represent the size and shape of the ROI, independently of the gray-level intensity distribution. Both “original_shape_MajorAxisLength,” which was calculated by using the largest principal component, and “original_shape_SurfaceArea,” which indicated the total surface area of the ROI, were related to the tumor size. From the first-order class, the most frequently selected radiomic feature was “original_firstorder_Uniformity,” which indicated homogeneity of the ROI intensity. For the texture and wavelet classes, the most frequently selected radiomic features were “gldm_DependenceNonUniformity,” “glrlm_GrayLevelNonUniformity,” and “glrlm_RunLengthNonUniformity.” These features measure similarity of gray-level patterns in the ROI. For “gldm_DependenceNonUniformity” which measured the similarity in dependency throughout the image, a lower value indicated more heterogeneity in the ROI and poorer prognosis. The “glrlm_GrayLevelNonUniformity” feature measured the similarity in gray-level intensity in the image, with a lower value indicating more tumor homogeneity. Similarly, the “glrlm_RunLengthNonUniformity” feature measured the similarity in run lengths throughout the images, with a lower value indicating more heterogeneity in the tumor. Most of the selected features in texture and wavelet classes reflected the pixel intensity distribution of ROI, which can be observed with the naked eye in tumors with poor prognoses (31). Apart from radiomic features, clinical features such as age and pretreatment plasma EBV DNA level were also frequently selected by the backward feature elimination process. This finding is in concordance with those of previous studies which have explored the correlation between age and disease prognosis in patients with NPC. It has been reported that younger patients had a better prognosis than older patients (36–39), and that plasma EBV DNA levels were linked to NPC early identification, prognostication, and treatment response (8–12).

Variability among observers remains a challenging issue in manual tissue segmentation, particularly for tumor delineation. In addition to intra-class correlation (ICC) analysis, which was performed to select consistent features for model construction, we also evaluated our model’s robustness. Specifically, we simulated the variation across observers based on tumor delineations by three radiation oncologists to generate synthetic radiomic feature values. The results showed that our models were robust with coefficient of variations less than 10% for both AUC and Harrell’s C-index.

There have been radiomics studies on NPC prognosis. MRI-based radiomics showed great promise for the prognosis of non-metastasis NPC patients (25, 27, 28, 40). Peng et al. (29) employed PET/CT-based radiomics as well as several clinical features to build a model to predict disease-free survival. They demonstrated that a radiomics model based on PET/CT outperformed a clinical model. In contrast to our findings, the clinical model produced better predictive performance than CT-based radiomic features. Furthermore, to the best of our knowledge, the only CT-based radiomics study on NPC was Zhu et al. (41), which used CT-based radiomic and clinical features including plasma EBV DNA level to predict local recurrences after IMRT in a cohort of 156 NPC patients from a hospital in China. Their results were also consistent with our findings even though we studied CT-based radiomics for OS, PFS, and DMFS prediction in non-Chinese patients.

Limitations of this study were that it was a retrospective study in a single center and that tumor delineation was based on CT images with MR images shown side by side. However, our findings serve as a proof of principle that CT-based radiomics is useful and we could identify important radiomic features that would be beneficial for future research. One of our future directions would be to construct a predictive model using radiomics from ROI drawn from CT images without support from MR images. Most importantly, the proposed predictive models should be further validated in a larger dataset from multiple centers or in patients who had induction chemotherapy followed by concurrent chemoradiation. The strength of this study includes a homogeneous group of patients who had been treated with IMRT concurrent with chemotherapy followed by adjuvant therapy. To the best of our knowledge, this is the first and largest study evaluating CT-based radiomics combined with clinical features on non-Chinese nasopharyngeal cancer patients to predict OS, PFS, and DMFS.

In conclusion, our study showed that CT-based Radiomic features, when used in conjunction with conventional clinical features, were able to improve the prognostic prediction performance for OS, PFS, and DMFS in NPC patients. This has a positive impact, especially for non-MRI institutes, on the screening of high-risk patients for aggressive therapeutic treatment strategies.

## Data Availability Statement

The raw data supporting the conclusions of this article will be made available by the authors, without undue reservation.

## Ethics Statement

The studies involving human participants were reviewed and approved by the Institutional Review Board, Faculty of Medicine, Chulalongkorn University, Bangkok, Thailand. The patients/participants provided their written informed consent to participate in this study.

## Author Contributions

SI: methodology, software, formal analysis, investigation, writing—original draft, visualization. YC: software, formal analysis. MV: software, formal analysis, investigation. SO: investigation. SK, AP, DK, and CC: data curation. SS: methodology, software, formal analysis, investigation. CL: conceptualization, methodology, resources, data curation. YR: conceptualization, methodology, formal analysis, investigation, resources, supervision, project administration, funding acquisition. All authors contributed to manuscript revision and read and approved the submitted version.

## Funding

This project was funded by the National Research Council of Thailand (NRCT) (Grant: NRCT5-RSA63001-14). Sararas Intarak was supported by the 100th Anniversary Chulalongkorn University Fund for Doctoral Scholarship.

## Conflict of Interest

The authors declare that the research was conducted in the absence of any commercial or financial relationships that could be construed as a potential conflict of interest.

## Publisher’s Note

All claims expressed in this article are solely those of the authors and do not necessarily represent those of their affiliated organizations, or those of the publisher, the editors and the reviewers. Any product that may be evaluated in this article, or claim that may be made by its manufacturer, is not guaranteed or endorsed by the publisher.

## References

[1] Sung H Ferlay J Siegel RL Laversanne M Soerjomataram I Jemal A. Global Cancer Statistics 2020: GLOBOCAN Estimates of Incidence and Mortality Worldwide for 36 Cancers in 185 Countries. CA Cancer J Clin (2021) 71(3):209–49. doi: 10.3322/caac.21660 33538338

[2] Tang LL Chen WQ Xue WQ He YQ Zheng RS Zeng YX. Global Trends in Incidence and Mortality of Nasopharyngeal Carcinoma. Cancer Lett (2016) 374(1):22–30. doi: 10.1016/j.canlet.2016.01.040 26828135

[3] Ferlay J Ervik M Lam F Colombet M Mery L Piñeros M. Global Cancer Observatory: Cancer Today(2020). Available at: https://gco.iarc.fr/today.

[4] Lee AWM Ma BBY Ng WT Chan ATC. Management of Nasopharyngeal Carcinoma: Current Practice and Future Perspective. J Clin Oncol (2015) 33(29):3356–64. doi: 10.1200/JCO.2015.60.9347 26351355

[5] Amin MB Greene FL Edge SB Compton CC Gershenwald JE Brookland RK. The Eighth Edition AJCC Cancer Staging Manual: Continuing to Build a Bridge From a Population-Based to a More “Personalized” Approach to Cancer Staging. CA Cancer J Clin (2017) 67(2):93–9. doi: 10.3322/caac.21388 28094848

[6] Ho JHC. Nasopharyngeal Carcinoma (Npc). In: Klein G Weinhouse S Haddow A, editors. Adv Cancer Res, vol. 15. New York: Academic Press (1972). p. 57–92. 10.1016/s0065-230x(08)60372-3 4333791

[7] Chan ATC Teo PML Johnson PJ. Nasopharyngeal Carcinoma. Ann Oncol (2002) 13(7):1007–15. doi: 10.1093/annonc/mdf179 12176778

[8] Chen YP Chan ATC Le QT Blanchard P Sun Y Ma J. Nasopharyngeal Carcinoma. Lancet (2019) 394(10192):64–80. doi: 10.1016/S0140-6736(19)30956-0 31178151

[9] Cao Y. EBV Based Cancer Prevention and Therapy in Nasopharyngeal Carcinoma. NPJ Precis Oncol (2017) 1(1):10. doi: 10.1038/s41698-017-0018-x 29872698 PMC5871899

[10] Tsao SW Tsang CM Lo KW. Epstein-Barr Virus Infection and Nasopharyngeal Carcinoma. Philos Trans R Soc Lond B Biol Sci (2017) 372(1732). doi: 10.1098/rstb.2016.0270 PMC5597737 28893937

[11] Wu L Li C Pan L. Nasopharyngeal Carcinoma: A Review of Current Updates. Exp Ther Med (2018) 15(4):3687–92. doi: 10.3892/etm.2018.5878 PMC5844099 29556258

[12] Young LS Dawson CW. Epstein-Barr Virus and Nasopharyngeal Carcinoma. Chin J Cancer (2014) 33(12):581–90. doi: 10.5732/cjc.014.10197 PMC4308653 25418193

[13] Lertbutsayanukul C Kannarunimit D Netsawang B Kitpanit S Chakkabat C Hansasuta P. Optimal Plasma Pretreatment EBV DNA Cut-Off Point for Nasopharyngeal Cancer Patients Treated With Intensity Modulated Radiation Therapy. Jpn J Clin Oncol (2018) 48(5):467–75. doi: 10.1093/jjco/hyy027 29522203

[14] Connor JPB Rose CJ Waterton JC Carano RAD Parker GJM Jackson A. Imaging Intratumor Heterogeneity: Role in Therapy Response, Resistance, and Clinical Outcome. Clin Cancer Res (2015) 21(2):249. doi: 10.1158/1078-0432.CCR-14-0990 25421725 PMC4688961

[15] Davnall F Yip CS Ljungqvist G Selmi M Ng F Sanghera B. Assessment of Tumor Heterogeneity: An Emerging Imaging Tool for Clinical Practice? Insights Imaging (2012) 3(6):573–89. doi: 10.1007/s13244-012-0196-6 PMC3505569 23093486

[16] Gillies RJ Kinahan PE Hricak H. Radiomics: Images Are More Than Pictures, They Are Data. Radiology (2016) 278(2):563–77. doi: 10.1148/radiol.2015151169 PMC4734157 26579733

[17] Lambin P Leijenaar RTH Deist TM Peerlings J de Jong EEC van Timmeren J. Radiomics: The Bridge Between Medical Imaging and Personalized Medicine. Nat Rev Clin Oncol (2017) 14(12):749–62. doi: 10.1038/nrclinonc.2017.141 28975929

[18] Kumar V Gu Y Basu S Berglund A Eschrich SA Schabath MB. Radiomics: The Process and the Challenges. Magn Reson Imaging (2012) 30(9):1234–48. doi: 10.1016/j.mri.2012.06.010 PMC3563280 22898692

[19] Lambin P Rios-Velazquez E Leijenaar R Carvalho S van Stiphout RG Granton P. Radiomics: Extracting More Information From Medical Images Using Advanced Feature Analysis. Eur J Cancer (2012) 48(4):441–6. doi: 10.1016/j.ejca.2011.11.036 PMC4533986 22257792

[20] Aerts HJ Velazquez ER Leijenaar RT Parmar C Grossmann P Carvalho S. Decoding Tumour Phenotype by Noninvasive Imaging Using a Quantitative Radiomics Approach. Nat Commun (2014) 5:4006. doi: 10.1038/ncomms5006 24892406 PMC4059926

[21] Wong AJ Kanwar A Mohamed AS Fuller CD. Radiomics in Head and Neck Cancer: From Exploration to Application. Transl Cancer Res (2016) 5(4):371–82. doi: 10.21037/tcr.2016.07.18 PMC6322843 30627523

[22] Giraud P Giraud P Gasnier A El Ayachy R Kreps S Foy JP. Radiomics and Machine Learning for Radiotherapy in Head and Neck Cancers. Front Oncol (2019) 9:174. doi: 10.3389/fonc.2019.00174 30972291 PMC6445892

[23] Zeng C Zhai T Chen J Guo L Huang B Guo H. Imaging Biomarkers of Contrast-Enhanced Computed Tomography Predict Survival in Oesophageal Cancer After Definitive Concurrent Chemoradiotherapy. Radiat Oncol (2021) 16(1):8. doi: 10.1186/s13014-020-01699-w 33436018 PMC7805131

[24] Luo H-S Huang S-F Xu H-Y Li X-Y Wu S-X Wu D-H. A Nomogram Based on Pretreatment CT Radiomics Features for Predicting Complete Response to Chemoradiotherapy in Patients With Esophageal Squamous Cell Cancer. Radiat Oncol (2020) 15(1):249. doi: 10.1186/s13014-020-01692-3 33121507 PMC7597023

[25] Du R Lee VH Yuan H Lam K-O Pang HH Chen Y. Radiomics Model to Predict Early Progression of Nonmetastatic Nasopharyngeal Carcinoma After Intensity Modulation Radiation Therapy: A Multicenter Study. Radiol: Artif Intell (2019) 1(4):e180075. doi: 10.1148/ryai.2019180075 33937796 PMC8017427

[26] Ming X Oei RW Zhai R Kong F Du C Hu C. MRI-Based Radiomics Signature Is a Quantitative Prognostic Biomarker for Nasopharyngeal Carcinoma. Sci Rep (2019) 9(1):10412. doi: 10.1038/s41598-019-46985-0 31320729 PMC6639299

[27] Li Q Wang T Huang Y Li Q Liu P Grimm R. Whole-Tumor Histogram and Texture Imaging Features on Magnetic Resonance Imaging Combined With Epstein-Barr Virus Status to Predict Disease Progression in Patients With Nasopharyngeal Carcinoma. Front Oncol (2021) 11(504). doi: 10.3389/fonc.2021.610804 PMC7986723 33767984

[28] Shen H Wang Y Liu D Lv R Huang Y Peng C. Predicting Progression-Free Survival Using MRI-Based Radiomics for Patients With Nonmetastatic Nasopharyngeal Carcinoma. Front Oncol (2020) 10:618. doi: 10.3389/fonc.2020.00618 32477932 PMC7235342

[29] Peng H Dong D Fang M-J Li L Tang L-L Chen L. Prognostic Value of Deep Learning PET/CT-Based Radiomics: Potential Role for Future Individual Induction Chemotherapy in Advanced Nasopharyngeal Carcinoma. Clin Cancer Res (2019) 25(14):4271–9. doi: 10.1158/1078-0432.CCR-18-3065 30975664

[30] Lertbutsayanukul C Prayongrat A Kannarunimit D Chakkabat C Netsawang B Kitpanit S. A Randomized Phase III Study Between Sequential Versus Simultaneous Integrated Boost Intensity-Modulated Radiation Therapy in Nasopharyngeal Carcinoma. Strahlenther Onkol (2018) 194(5):375–85. doi: 10.1007/s00066-017-1251-5 29302704

[31] van Griethuysen JJM Fedorov A Parmar C Hosny A Aucoin N Narayan V. Computational Radiomics System to Decode the Radiographic Phenotype. Cancer Res (2017) 77(21):e104–e7. doi: 10.1158/0008-5472.CAN-17-0339 PMC5672828 29092951

[32] Koo TK Li MY. A Guideline of Selecting and Reporting Intraclass Correlation Coefficients for Reliability Research. J Chiropr Med (2016) 15(2):155–63. doi: 10.1016/j.jcm.2016.02.012 PMC4913118 27330520

[33] Davidson-Pilon C. Lifelines: Survival Analysis in Python. J Open Source Softw (2019) 4:1317. doi: 10.21105/joss.01317

[34] Pedregosa F Varoquaux G Gramfort A Michel V Thirion B Grisel O. Scikit-Learn: Machine Learning in Python. J Mach Learn Res (2011) 12(85):2825–30.

[35] Stata Corp. Stata Statistical Software: Release 15. College Station, TX: StataCorp LLC (2017).

[36] Ou SHI Zell JA Ziogas A Anton-Culver H. Epidemiology of Nasopharyngeal Carcinoma in the United States: Improved Survival of Chinese Patients Within the Keratinizing Squamous Cell Carcinoma Histology. Ann Oncol (2007) 18(1):29–35. doi: 10.1093/annonc/mdl320 17060483

[37] Jiong L Berrino F Coebergh JWW. Variation in Survival for Adults With Nasopharyngeal Cancer in Europe, 1978–1989. Eur J Cancer (1998) 34(14):2162–6. doi: 10.1016/S0959-8049(98)00322-0 10070282

[38] Yeh S-A Tang Y Lui C-C Huang Y-J Huang E-Y. Treatment Outcomes and Late Complications of 849 Patients With Nasopharyngeal Carcinoma Treated With Radiotherapy Alone. Int J Radiat Oncol Biol Phys (2005) 62(3):672–9. doi: 10.1016/j.ijrobp.2004.11.002 15936544

[39] Yi J-l Gao L Huang X-d Li S-y Luo J-w Cai W-m. Nasopharyngeal Carcinoma Treated by Radical Radiotherapy Alone: Ten-Year Experience of a Single Institution. Int J Radiat Oncol Biol Phys (2006) 65(1):161–8. doi: 10.1016/j.ijrobp.2005.12.003 16542792

[40] Zhang B Tian J Dong D Gu D Dong Y Zhang L. Radiomics Features of Multiparametric MRI as Novel Prognostic Factors in Advanced Nasopharyngeal Carcinoma. Clin Cancer Res (2017) 23(15):4259–69. doi: 10.1158/1078-0432.CCR-16-2910 28280088

[41] Zhu C Huang H Liu X Chen H Jiang H Liao C. A Clinical-Radiomics Nomogram Based on Computed Tomography for Predicting Risk of Local Recurrence After Radiotherapy in Nasopharyngeal Carcinoma. Front Oncol (2021) 11:637687. doi: 10.3389/fonc.2021.637687 33816279 PMC8012724

